# Bovine Papillomatosis Hiding a Zoonotic Infection: Epitheliotropic Viruses in Bovine Skin Lesions

**DOI:** 10.3390/pathogens9070583

**Published:** 2020-07-17

**Authors:** Laura Gallina, Federica Savini, Sabrina Canziani, Matteo Frasnelli, Antonio Lavazza, Alessandra Scagliarini, Davide Lelli

**Affiliations:** 1 Dipartimento di Scienze Mediche Veterinarie, Alma Mater Studiorum Università di Bologna, 40064 Ozzano Emilia, Italy; federica.savini3@unibo.it; 2 Istituto Zooprofilattico Sperimentale della Lombardia e dell’Emilia Romagna “Bruno Ubertini” (IZSLER), 25124 Brescia, Italy; sabrina.canziani@izsler.it (S.C.); matteo.frasnelli@izsler.it (M.F.); antonio.lavazza@izsler.it (A.L.); davide.lelli@izsler.it (D.L.); 3 Dipartimento di Medicina Specialistica, Diagnostica e Sperimentale, Alma Mater Studiorum Università di Bologna, 40138 Bologna, Italy; alessand.scagliarini@unibo.it

**Keywords:** bovine, parapoxvirus, papillomavirus, herpesvirus, skin, co-infection, zoonosis

## Abstract

We describe two cases of skin co-infections with epitheliotropic viruses, detected in two cattle during lumpy skin disease (LSD) surveillance in northern Italy. A diagnostic protocol including different molecular methods as well as negative staining electron microscopy was applied to detect the most common viral agents belonging to the family *Papillomaviridae*, *Poxviridae* and *Herpesviridae* which cause skin diseases in cattle. Two specimens were collected from cases clinically diagnosed as papillomatosis and pseudo-LSD. Both skin lesions were shown to harbor more than one viral species. This case report shows, for the first time, co-infection of zoonotic parapoxvirus with bovine papillomavirus and herpesvirus in skin lesions of cattle. In particular, the simultaneous presence of virions morphologically referable to parapoxvirus and papillomavirus confirms that the replication of both viruses in the same lesion can happen and the so-called papillomatosis can bear zoonotic viruses.

Skin represents the main barrier between external, potentially aggressive agents and inner, more vulnerable structures and represents a complex system harboring various populations of micro-organisms known as skin microbiota. Hyperproliferative skin lesions affecting the tissue and mucosa of cattle can be caused by bovine papillomavirus (BPVs), non-enveloped double stranded DNA viruses belonging to the *Papillomaviridae* family. Despite the benign characteristics of the lesions, in some cases, bovine papillomatosis can dramatically reduce production performance when lesions are spread throughout the body, involving teats and udders. We have previously shown that the so-called “papillomatosis” can be the result of multiple infections with epitheliotropic viruses, including zoonotic poxviruses [1,2].

Poxvirus and papillomavirus have been simultaneously detected in the skin lesions of cattle [3,4], birds [5,6] and humans [7], suggesting that these viruses can complete a replication cycle in the same lesions.

Here we describe two further cases of skin co-infections with epitheliotropic viruses that have been detected during a surveillance program for the viral cutaneous diseases of cattle implemented in Italy following the onset of lumpy skin disease (LSD) in some eastern European countries.

A diagnostic protocol, specifically set up to detect the most common viral agents causing skin diseases in cattle, was applied to cutaneous lesions collected from cattle involved in clinical cases reported in northern Italy. Briefly, skin lesions were tested for the presence of BPVs, bovine herpesviruses, parapoxviruses, orthopoxviruses and capripoxviruses using different techniques, combining molecular methods [1,8,9,10,11,12,13,14] as well as negative staining electron microscopy (nsEM) [15]. In Table 1, the virological diagnostic methods used in the study are reported.

The first clinical case (#7) was identified on February 2018 at the slaughterhouse in a 20 months-old Charolaise cow in which cutaneous proliferative lesions were observed (Figure 1) and pathological skin samples were collected. Anamnesis reported a story of similar lesions in cattle from the same farm; the lesions appeared periodically but especially during the period December 2017–April 2018.

The second clinical case (#1616) was identified during an outbreak of pseudo-LSD in a Fresian dairy farm during summer 2018. The 100-day-old calf presented lumps, scattered circular areas of alopecia and surface crust formation on the back, neck, shoulders and head (Figure 2).

In both cases, nsEM, RCA isothermal amplification, PCRs and sequencing of the amplification products were performed on pathological skin samples in order to identify the most common pathogens associated with cattle skin lesions, as well as bovine papillomavirus’ unknown circular genomes.

In the samples from case (#7), nsEM identified the simultaneous presence of parapoxvirus mature virions and non-enveloped icosahedral particles morphologically referable to papillomavirus (Figure 3A).

RCA followed by digestion with BamHI, HindIII and KpnI revealed the presence of BPV-12 while sequencing of the PCR products showed a co-infection with a zoonotic parapoxvirus and another bovine papillomavirus. The sequence obtained by the PCR specific for parapoxvirus (GenBank accession number: MT68213) showed a 99.81% identity to bovine papular stomatitis virus (BPSV) (GenBank accession number: MK285566). The presence of a second papillomavirus co-infecting the skin lesion was confirmed by the sequences of the E5 open reading frame (ORF) (GenBank accession number: MT682132) and partial L1 ORF (GenBank accession number: MT682133), which showed 100% identity to BPV-1 (GenBank accession number: MF384289.1).

Previous reports have identified the co-existence of parapox and orthopoxvirus particles in skin lesions of cattle by EM [16]. In humans, the simultaneous infection with human papillomavirus and poxvirus has shown the epidermis and follicle as the target area of viral infection [17]. To our knowledge, this is the first time that parapoxvirus and papillomavirus particles have been simultaneously detected by nsEM. This finding suggests that the production of infectious viral particles as a consequence of papillomavirus and parapoxvirus replication can occur. The rare co-existence of epitheliotropic viruses within epidermal lesions can take place since different cellular compartments and target cells can be used for these viruses to exert their pathogenic role with no interference. Poxviruses are known to exert host response evasion through virulence genes expressing immunomodulatory factors that may enhance the survival of papillomavirus in dual infections as reported by Payne and collaborators [7].

In samples from case (#1616), nsEM showed the presence of herpesviral particles (Figure 3B) subsequently identified as BoHV2 by PCR and sequencing (GenBank accession number: MT682134).

However, the protocol used for diagnosis also included a pan-parapoxvirus PCR followed by Sanger sequencing that allowed us to detect the zoonotic pseudocowpoxvirus (PCPV) (Genbank accession number: MT682135). Genomic amplification did not allow us to identify any bovine papillomavirus (BPVs). This finding further proves that virus co-infections are also possible between herpes and parapoxviruses. The definitive PLSD diagnosis for sample (#1616) was based on nsEM and sequencing of BoHV2, but the detection of PCPV DNA cannot be ignored since parapoxvirus should always be included in differential diagnosis. The impossibility of identifying PCPV by electron microscopy can be the result of a low viral load. On the other hand, the presence of PCPV DNA in the pathological samples led us to speculate that the virus may use such lesions as reservoirs to spread and persist in the environment through the scab material produced by other epitheliotropic viruses.

Our study confirms that cattle skin lesions may host diverse viral agents including zoonotic poxviruses. This is particularly important considering that all the parapoxviruses are responsible for common occupational zoonoses and humans can be infected after direct contact with affected cattle or indirectly through a contaminated environment as viruses survive for long periods when protected by organic material.

## Figures and Tables

**Figure 1:**
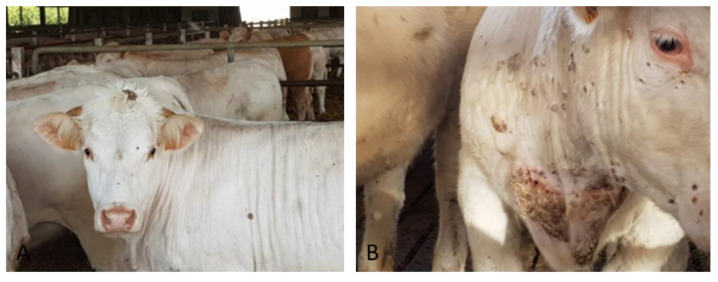
Skin proliferative lesion typical of bovine papillomatosis identified in Charolaise breeding cow sample (#7) (**A**) papilloma on the head (**B**) neck and shoulder.

**Figure 2:**
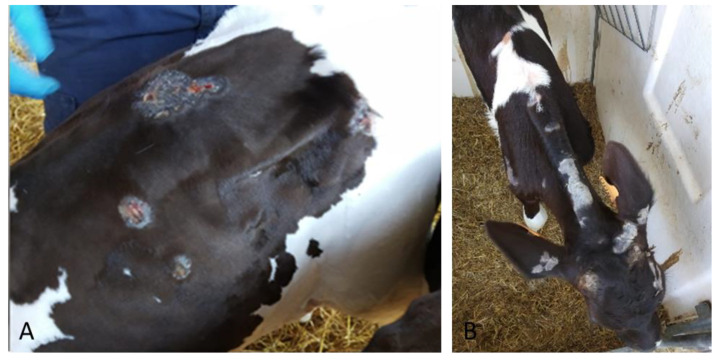
Skin lesion identified in a Fresian dairy farm sample (#1616). The animal presented lumps, (**A**) scattered circular areas of alopecia and surface crust formation on back, (**B**) neck, shoulders and head.

**Figure 3:**
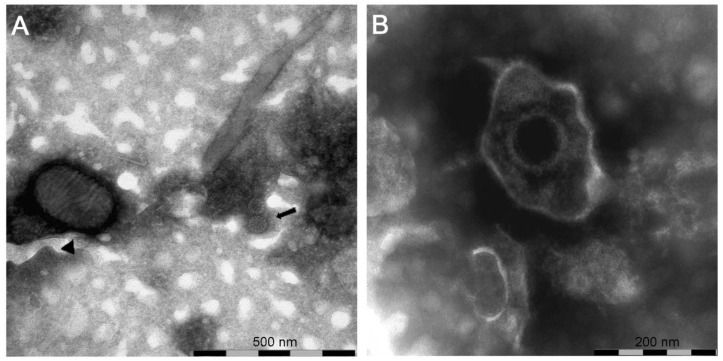
Negative-staining electron microscopy of: (**A**) sample (#7) showing the presence of a virions morphologically related to parapoxvirus ▲ and papillomavirus 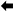; (**B**) sample (#1616) showing the presence of a virion morphologically related to herpesvirus.

**Table 1:** Virological diagnostic methods used in the study.

Viral Target	Method	References
Pan papillomavirus (partial L1 ORF)	End-point PCR	Forslund et al., 1999 [8]
Bovine papillomavirus type 1–2 (complete E5 ORF)	End-point PCR	Brandt et al., 2008 [9]
Bovine papillomavirus type 3, 4, 6, 9, 10 (partial L1 ORF)	End-point PCR	Brandt et al., 2011 [10]
Bovine papillomavirus (typing using restriction endonucleases)	RCA (rolling circle amplification).	Rector et al., 2004 [11]
Herpersvirus (partial DPOL gene)	PCR/RFLP	De Giuli et al., 2002 [12]
Parapoxvirus (partial ORF11)	End-point PCR	Inoshima et al., 2000 [13]
Orthopoxvirus (partial rpo18 ORF)	End-point PCR	Scagliarini et al., 2016 [1]
Capripoxvirus (partial ORF074)	Real-time PCR	Stubbs et al., 2012 [14]
Wide range of viral targets	Negative staining electron microscopy (nsEM)	Lavazza et al., 1990 [15]

ORF: open reading frame; DPOL: DNA-dependent DNA polymerase gene; rpo18: 18-kDa subunit of the RNA polymerase.
